# The Rapid Non-Destructive Differentiation of Different Varieties of Rice by Fluorescence Hyperspectral Technology Combined with Machine Learning

**DOI:** 10.3390/molecules29030682

**Published:** 2024-02-01

**Authors:** Zhiliang Kang, Rongsheng Fan, Chunyi Zhan, Youli Wu, Yi Lin, Kunyu Li, Rui Qing, Lijia Xu

**Affiliations:** 1 College of Mechanical and Electrical Engineering, Sichuan Agriculture University, Ya’an 625000, China; kangzhiliang@sicau.edu.cn (Z.K.); fanrongsheng@stu.sicau.edu.cn (R.F.); zhanchunyi@stu.sicau.edu.cn (C.Z.); 2021217008@stu.sicau.edu.cn (Y.W.); linyi@stu.sicau.edu.cn (Y.L.); likunyu@stu.sicau.edu.cn (K.L.); 2023217014@stu.sicau.edu.cn (R.Q.); 2 Sichuan Research Center for Smart Agriculture Engineering Technology, Ya’an 625000, China

**Keywords:** rice, fluorescence hyperspectral technology, variety classification, non-destructive

## Abstract

A rice classification method for the fast and non-destructive differentiation of different varieties is significant in research at present. In this study, fluorescence hyperspectral technology combined with machine learning techniques was used to distinguish five rice varieties by analyzing the fluorescence hyperspectral features of Thai jasmine rice and four rice varieties with a similar appearance to Thai jasmine rice in the wavelength range of 475–1000 nm. The fluorescence hyperspectral data were preprocessed by a first-order derivative (FD) to reduce the background and baseline drift effects of the rice samples. Then, a principal component analysis (PCA) and t-distributed stochastic neighborhood embedding (t-SNE) were used for feature reduction and 3D visualization display. A partial least squares discriminant analysis (PLS-DA), BP neural network (BP), and random forest (RF) were used to build the rice classification models. The RF classification model parameters were optimized using the gray wolf algorithm (GWO). The results show that FD-t-SNE-GWO-RF is the best model for rice classification, with accuracy values of 99.8% and 95.3% for the training and test sets, respectively. The fluorescence hyperspectral technique combined with machine learning is feasible for classifying rice varieties.

## 1. Introduction

Rice, corn, and wheat are the world’s three major food crops [1], with rice being one of the most dominant staple foods in many countries, especially in some Asian countries [2]. Since 1960, thousands of rice varieties have appeared in the world [3]. With the improvement in the population’s living standards, the quality requirements for the daily consumption of rice are also increasing [4]; rice should taste good and flavorful and be nutritious [4]. Rice provides the necessary protein and energy and calcium, iron, zinc, selenium, potassium, and other mineral elements for the human body [4]. Because of its rich nutritional value and good flavor and texture, rice occupies an essential position in the staple food market and other processed foods [5]. At present, there are many varieties of rice available in the market [6]. However, the quality and nutritional value of different rice varieties are different [6], and there is also a significant difference in the selling price. Among them, Thai jasmine rice’s appearance, good quality, and fragrant smell are loved by consumers worldwide [7]. However, due to its limited production, the mixing of the expensive Thai jasmine rice with ordinary white rice is becoming an increasingly problematic phenomenon [8]. The malpractice creates unfair economic interests, undermining the consumer’s trust in the producer [9], affecting the consumer’s consumption of taste, and causing severe harm to the health of the body [10]. At the same time, rice adulteration has become an urgent problem in the food industry [9]. Therefore, research on rice variety detection technology has become a hot research topic in the field of food safety at present [11]. Developing an effective tool for consumers, producers, and retailers to identify rice varieties is necessary.

**Figure 1:**
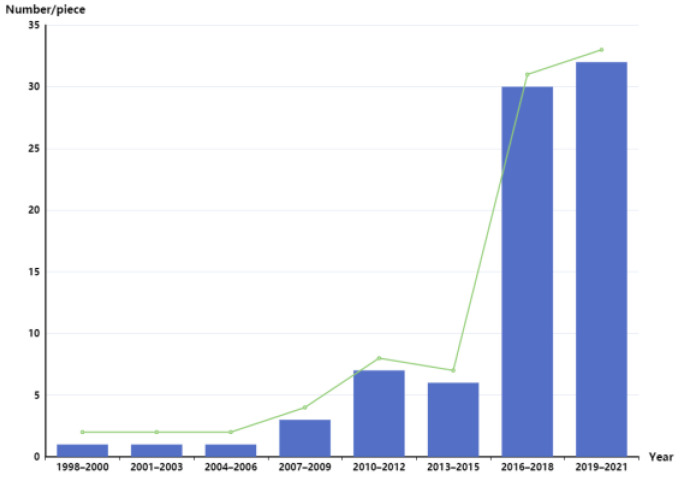
Classification of domestic and foreign rice varieties.

In recent years, research on rice variety identification has been carried out at home and abroad [12]. As shown in Figure 1, from 1998 to 2005 [13], people’s awareness of rice consumption safety was relatively poor [13], and there was less research on rice variety identification and authenticity. Since 2006, the research on variety identification and the authenticity of rice has gradually increased [14], mostly between 2012 and 2015. Because of the frequent occurrence of rice quality problems and safety events in this period [15], countries have established agricultural management regulations and used the law to clarify the traceability system of rice [15]. People’s awareness of the safety of rice consumption increased yearly, and more and more researchers have begun to research rice varieties and their authenticity [10].

Traditional rice variety identification methods include sensory evaluation [12], physical and chemical testing [12], spectroscopic methods [8], and electronic tongue and nose technology [12]. The sensory evaluation method is the most frequently used rice variety testing method, mainly including direct comparison and coloring [16]. Professionals use sensory evaluation to score rice’s taste, color, and texture, and subjective factors easily influence it [12]. Standard chemical detection methods include GC-MS [10], LC-MS [10], and GC-IMS [10], which can qualitatively and quantitatively detect the chemical components of rice, such as straight-chain starch content, fatty acids, proteins, and starch [10]. However, it requires a complicated sample pretreatment and professional operation [10]. Standard physical testing methods include texture and rapid viscosity analyzers [10], which can create accurate expressions by testing rice’s hardness, viscosity, cohesion, and resilience to evaluate its edible quality objectively [10]. All of the above methods require sample pretreatment, sample structure disruption, and specialized personnel. Raman and near-infrared spectroscopy have the drawbacks of cumbersome sample preparation [8], being time-consuming, and non-destructive detection. An electronic tongue [17] and electronic nose [18] can simulate those of mammals to identify the characteristics of the sample through taste and smell. This method obtains qualitative and quantitative information from the sample by making non-specific responses to the relevant chemicals and analyzing the responses through appropriate pattern recognition procedures [10]. However, this method also suffers from the problem of being affected by the environment and destroying the structure of the rice sample [10]. The above traditional rice variety identification methods are time-consuming, cumbersome, and destructive [10]. They cannot meet the growing demand for rapid, non-destructive testing in the supervision and distribution of the rice market industry. Therefore, because of the shortcomings of the above methods, it is urgent to develop a method for rice variety identification that is highly accurate, rapid, efficient, and low-cost.

Spectral imaging technology [19], as a non-contact rapid detection means, has been widely used in research related to rice detection [19]. Among them, fluorescence hyperspectral [20] imaging technology combines the advantages of hyperspectral technology and digital imaging technology, which can effectively make up for the shortcomings of traditional rice identification methods. Moreover, fluorescence hyperspectral imaging is a non-destructive spectral analysis technique [20], which provides a new way of thinking for the non-destructive testing of rice. This technology excites the electrons in the material to be measured by a laser or other light sources, causing the electrons to jump from a low to a high energy level [21]. When the electrons are at a high energy level, they immediately return to a low one, releasing energy [21]. This energy is emitted in the form of fluorescence, from which the spectral camera captures the image information and obtains a fluorescence hyperspectral image of the sample [21]. The principle of fluorescence hyperspectral imaging is similar to that of hyperspectral imaging [19]. However, the difference is that, in fluorescence hyperspectral imaging, the spectral camera receives the fluorescence intensity emitted by the sample’s fluorescent substance rather than the sample’s reflected light [20]. This technique has a short detection time and does not cause damage to the sample itself [21]. Therefore, this study combines fluorescence hyperspectral imaging with rice variety identification, which has tremendous research potential.

Fluorescence hyperspectral technology combined with machine learning [22] has yet to be used to explore rice variety identification. In this study, Thai jasmine rice that was sold in the market and four other common rice species with a similar appearance to Thai jasmine rice are selected. The purpose of this study is to use a novel fluorescence hyperspectral imaging device to obtain the fluorescence hyperspectral data of the five types of rice and then analyze and process the fluorescence hyperspectral data using algorithms in the field of machine learning in order to establish a rice variety identification model for distinguishing the authenticity of Thai jasmine rice. This study provides a reference method and theoretical basis for the research of maintaining Thai jasmine rice’s market order, protecting consumers’ rights and interests, and safeguarding Thai jasmine rice’s food safety. It also provides a rapid and non-destructive testing method for other food safety identification fields.

## 2. Results and Discussion

### 2.1. Characterization of Fluorescence Hyperspectral Imaging

**Figure 2:**
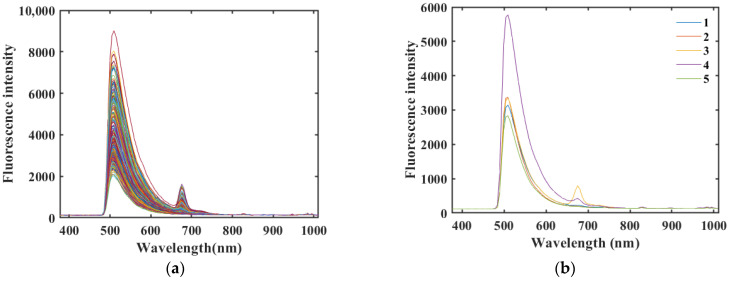
(**a**) Raw spectra of the five rice samples. (**b**) Raw average spectra of the five rice samples (1—Northeast Wuchang rice, 2—Northeast long-grain rice, 3—Thai jasmine rice, 4—Sichuan Meishan rice, 5—Shaanxi Hanzhong rice; the same below).

A fluorescence hyperspectral imaging device was used to collect spectral data, and a total of 550 (5 species × 110 samples) rice samples were obtained. The raw fluorescence hyperspectral values of the rice samples are shown in Figure 2 (in the pictures in this section, 1 represents Northeast Wuchang rice, 2 represents Northeast long-grain rice, 3 represents Thai jasmine rice, 4 represents Sichuan Meishan rice, and 5 represents Shaanxi Hanzhong rice). The fluorescence hyperspectral curve trend, peak, and trough positions are the same for the five different rice samples in the spectral range of 376–1000 nm [23], but the reflectance values differ. This indicates that the internal chemical composition of the rice is the same, but the content of each component is different [23].

According to Figure 2b, each of the five types of rice showed different fluorescence hyperspectral waveforms in the range of 480–600 nm, and all of them exhibited a fluorescence characteristic peak at 509 nm, which was related to saturated fatty acids, the main component in rice. In particular, Thai jasmine rice exhibited the highest peak at 675 nm compared to the four ordinary Chinese rice varieties, which was related to chlorophyll in rice. These components are factors that affect the quality of rice. These factors are influenced by nitrogen fertilizer application, the production environment, and the cultivation environment (temperature). Among them, the four ordinary Chinese rice varieties were grown at higher latitudes than Thai jasmine rice (15–18° N), which was grown in the tropics. The reason for the trend of fluorescence intensity in the above rice samples is similar to that reported by Min-Jee Kim et al. [23]. This further validates the accuracy of the results of this study. The internal differences between Thai jasmine rice and Chinese rice resulted in different fluorescence intensities in the fluorescence spectra, providing a theoretical basis for identifying pure Thai jasmine rice, which is difficult to differentiate with the naked eye, and ordinary rice, which has a similar appearance, in the market.

### 2.2. Fluorescence Hyperspectral Data Preprocessing

**Figure 3:**
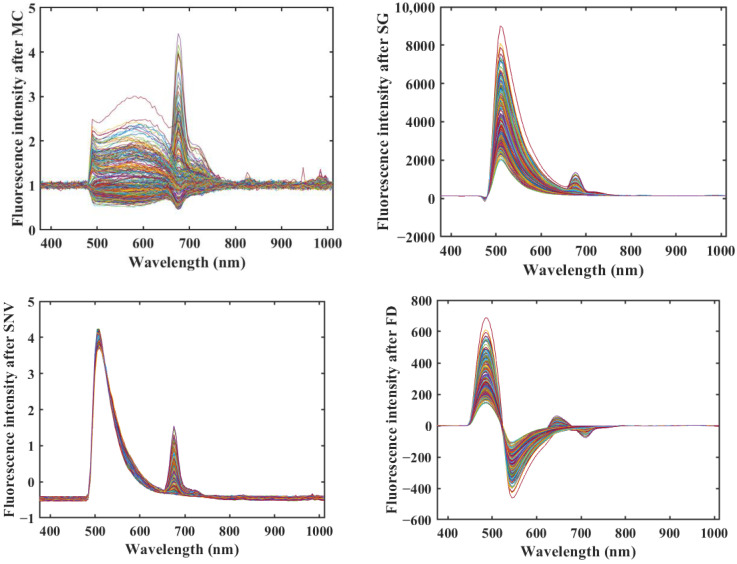
Spectra of the rice samples after different pretreatments.

In the process of fluorescence hyperspectral data acquisition, due to the influence of environmental factors, there is a certain amount of noise in the acquired fluorescence hyperspectral data, which adversely affects the performance of the final modeling. Therefore, fluorescence hyperspectral data need to be preprocessed before modeling [22,24]. Figure 3 shows the fluorescence hyperspectral curves after MC, SG, SNV, and FD preprocessing.

**Figure 4:**
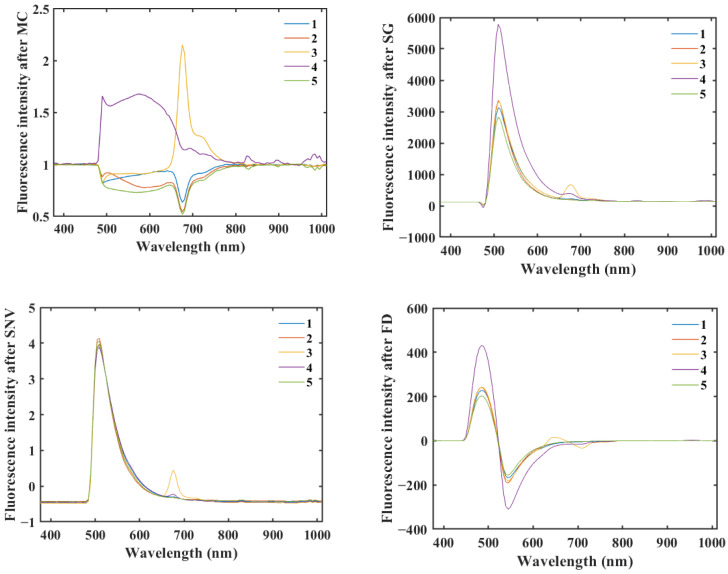
Mean spectra of the rice samples after different pretreatments.

Figure 2, Figure 3 and Figure 4 show that all three methods, SG, SNV, and FD, performed better on the rice sample data preprocessing than the original data, and the MC preprocessing method produced more interference. SG and SNV performed roughly the same in eliminating the solid particle size and surface scattering and in highlighting helpful information [25]—the difference between the fluorescence hyperspectral curves after SG and SNV processing is not apparent. However, after the FD preprocessing of the original fluorescence hyperspectral curves, the baseline translation changes can be corrected [26], and the differences in the fluorescence hyperspectral curves of the different rice varieties are improved, which can reflect the fluorescence hyperspectral characteristics of the samples in more detail.

We compared the different preprocessed data using various machine learning classification models and obtained the classification accuracy through the ten-fold cross-validation of the models [27]. We divided the rice fluorescence hyperspectral dataset into ten parts and took turns using nine parts as the training dataset and one part as the test dataset, with the average accuracy of 10 results from the test set as an estimate of the algorithm accuracy. Table 1 shows the accuracy of the various preprocessing methods (MC, SG, SNV, and FD) of the raw fluorescence hyperspectral data for the test set in different classification models.

As shown in Table 1, the overall classification accuracy of the models with raw fluorescence hyperspectral data (RAW) without preprocessing was the lowest for the same model. In contrast, the overall classification accuracy of the models with preprocessing was improved. Among them, the FD and SNV test sets had the best accuracy for all classification models. The test sets obtained from the RF classification model were higher than the preprocessing methods used for the other models. Among all the preprocessing methods, MC had the worst preprocessing of data, which affected the model’s accuracy. Nevertheless, RF was 81%, which had the best accuracy on the RF classification model. From Table 1 and Figure 2, Figure 3 and Figure 4, the preprocessed methods applied for use on the classification model were feasible and improved the accuracy of the classification model. After comparison, two preprocessing methods, SNV and FD, were used to conduct the subsequent research.

### 2.3. Feature Downscaling and Selection

**Figure 5:**
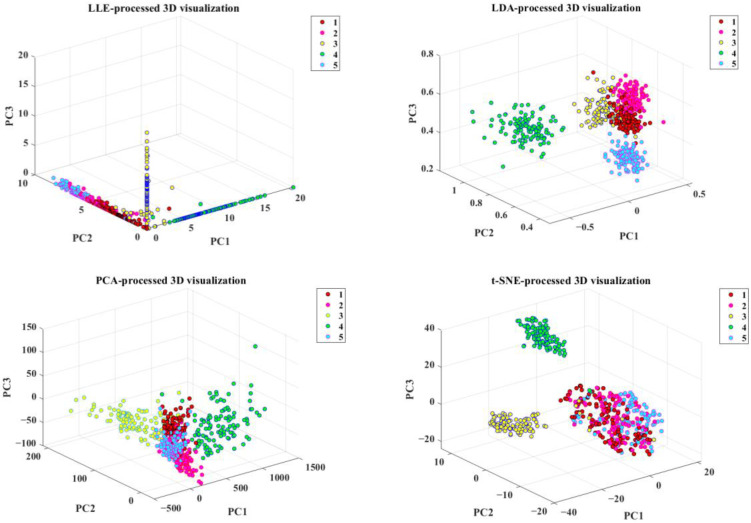
Three-dimensional visualization of the rice samples after dimensionality reduction processing using different features.

After preprocessing, a large amount of information is still not related to the data [20]. If adequate information is not further extracted, the high-dimensional data affect the accuracy and robustness of the model [20]. In this paper, LLE, LDA, PCA, and t-SNE were selected for feature dimensionality reduction selection, which can reduce the dimensionality of the fluorescence hyperspectral data and eliminate the part of the information that overlaps with each other in the data information [28]. By transforming the original fluorescence hyperspectral data variables, a smaller number of new variables become linear combinations of the original variables; moreover, the new variables can maximally characterize the original variables’ data structure features and retain the original information data [28]. This study projected rice samples onto a three-dimensional spatial coordinate system composed of the score matrix’s first three feature principal components (PC1, PC2, and PC3). We visually displayed them as three-dimensional components, as shown in Figure 5.

As seen in Figure 5, all four feature downscaling methods can display the five types of rice in the form of “clusters”. The rice sample points reduced to three dimensions by the LLE method are considerably overlapped in the three-dimensional space. In the rice samples processed by LDA, the sample points of five kinds of rice are interspersed with each other, and the sample points of the five kinds of rice are very close to each other in the three-dimensional space, which does not clearly distinguish the five kinds of rice. The five clustering features in the rice samples processed using PCA are more pronounced and can be distinguished. After using t-SNE to process the rice samples, the five clustering characteristics are apparent, and the five clusters of data points are farther apart; especially, the Thai jasmine rice and Sichuan Meishan rice clustering effect is the best, and the remaining three varieties of the clusters only have a small amount of crossover among the clusters. This is because the t-SNE dimensionality reduction method can replace the Gaussian distribution in the low-dimensional space with a t-distribution, and the long-tailed nature of the t-distribution [29] (low in the center and high and long in the tails [29]) separates the sample points of the five varieties of rice more obviously. Yan Hu et al. [20] used t-SNE and PCA to construct a tea variety classification model through three-dimensional visualization. The model’s classification performance based on t-SNE was better than that of the model based on PCA, which further indicated that t-SNE had a more substantial applicability to nonlinear high-dimensional data. From the visualization effect of the rice samples in Figure 5, both PCA and t-SNE can retain most of the information in the spectral curve, effectively reducing the dimensionality of the rice sample spectral data. However, the classification boundary between the two is unclear, and it is necessary to establish relevant rice classification models for further identification to see if the accuracy of the model construction can be improved.

### 2.4. Modeling Analysis after Preprocessing and Feature Dimensionality Reduction Processing

The accuracy of the building classification prediction models after using different preprocessing methods and feature dimensionality reduction processing methods is shown in Table 2. The dataset for training the model in Table 2 is the dataset segmented using the randomized segmentation method in machine learning, where the training dataset is used for model calibration and the test dataset is used for external validation. This division method references the division method of the training and test sets in reference [30]. The distribution of the dataset using this method is relatively uniform; the distribution of the training set and the test set overlap, and the samples in the test set contain the features of all the samples in the training set, which ensures the reliability of the rice sample classification.

The following can be seen from the comparison of Table 1 and Table 2:

(a) In this study, a classification model was used to test the accuracy of the best rice model. After preprocessing and feature dimensionality reduction, the classification model accuracy was higher than that modeled using raw fluorescence hyperspectral data. This is because, by preprocessing, the spectral noise can be removed from the spectral curve as much as possible, highlighting the valuable information of the spectrum [31]. Then, after processing by feature dimensionality reduction, the spectral data dimensions are reduced to reduce further the influence of spectral noise, which reduces the amount of data and the influence of useless data [32]. After preprocessing and feature dimensionality reduction processing, the modeling accuracy is somewhat improved, and the robustness is enhanced.
(b) Among all the feature dimensionality reduction processing methods, the accuracy of t-SNE in the RF modeling was dramatically improved. Theoretically, PCA is a matrix decomposition technique involving multiple conditional probabilities and gradient descent calculations [33], while t-SNE is a probabilistic method [34]. In other classification models, t-SNE also has a higher accuracy than PCA.
(c) The accuracy of the experimental results showed significant differences in the effects of the different models. The accuracy of RF was improved on t-SNE and PCA and was not affected by its underlying evaluator. When the data were reduced to three dimensions by FD-t-SNE-RF, the model was more accurate than the other preprocessing methods. RF had a higher classification accuracy than BP and PLS-DA. RF had a significant advantage in the classification of rice in this study. After using RF, the accuracy of different preprocessing and feature processing methods was higher than the other two classification models. Especially in FD-t-SNE-RF, the accuracies of the training and test sets were 99.7% and 93.3%, respectively.

**Figure 6:**
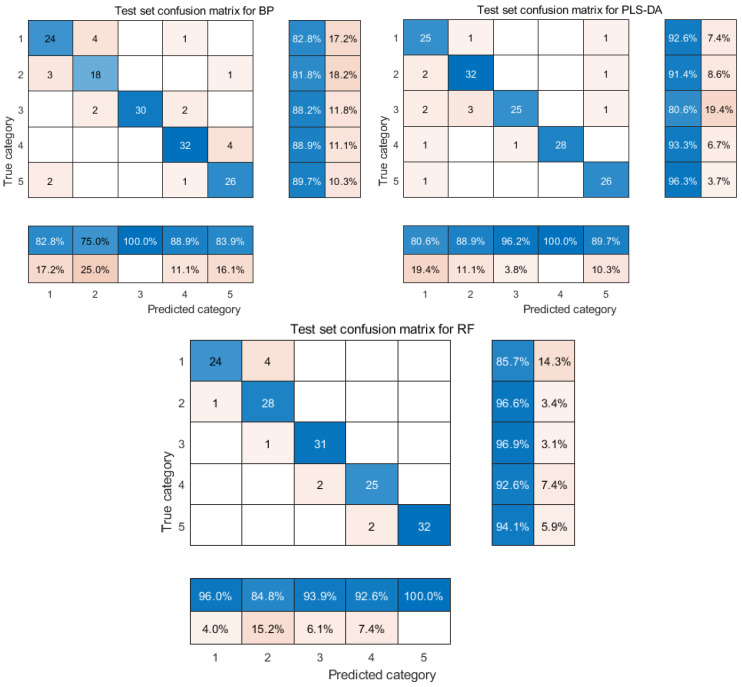
Confusion matrix of the classification model after the FD-t-SNE treatment (diagonal numbers are the number of correctly classified rice samples).

**Figure 7:**
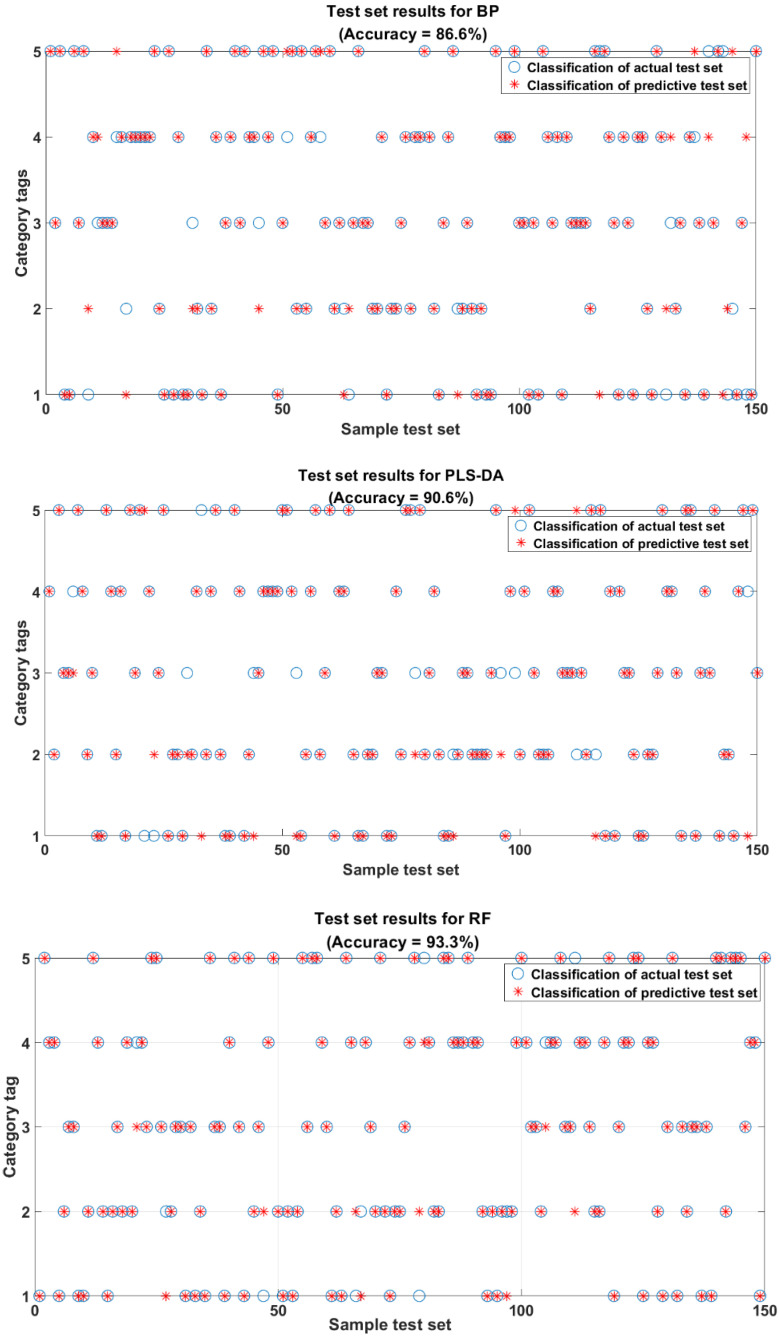
Scatterplot of the classification model after FD-t-SNE processing.

In order to validate the modeling performance, 150 unknown rice samples were selected as the test set by the random division method [30]. Figure 6 shows the confusion matrix [34] of the classification model for the prediction set of 150 samples after FD-t-SNE processing. The rightmost two columns of data represent the precision of the correct classification and the precision [34] of the incorrect classification for each category. The two columns of data at the bottom represent the recall [34] of classification for each category. From Figure 6, it can be seen that the classification model of PLS-DA in the five categories of precision from top to bottom are 92.6%, 91.4%, 80.6%, 93.3%, and 96.3%, in which the precisions of four categories are higher than those of the BP classification model. From Figure 6 and Figure 7, it can be seen that, out of the 150 samples, PLS-DA correctly identifies 136 samples with an overall accuracy of 90.6%; BP can only correctly recognize 130 rice samples with an overall accuracy of 86.6%. The overall performance of the PLS-DA classification model is better than that of the BP classification model. Although PLS-DA has a better precision of three out of five categories than the RF classification model, the overall accuracy of RF is 93.3% higher than that of the PLS-DA classification model. Therefore, the RF classification model was chosen for the rice classification modeling in this study.

As it can be seen from Figure 6, in the RF classification model, 2 (Northeast long-grain aromatic rice) and 3 (Thai jasmine rice) each had one error, misclassified as 1 (Northeast Wuchang rice) and 2 (Northeast long-grain aromatic rice), respectively. The 4 (Sichuan Meishan rice) and 5 (Shaanxi Hanzhong rice) varieties each had two errors, misclassified as Thai jasmine rice and Sichuan Meishan rice, respectively. The Northeast Wuchang rice showed four errors, misclassified as Northeast long-grain fragrant rice, the most misclassifications among the five categories. This may be because Northeast Wuchang rice and Northeast long-grain fragrant rice are produced in the Northeast Heilongjiang region. As shown in Figure 1, the fluorescence hyperspectral information of these two types of rice is relatively close to each other [23], which makes the model classification results biased. The precision of the rice classification of the remaining four categories is greater than 92%. Overall, the RF classification model can accurately classify rice varieties. Liu Wei et al. [30] achieved the best discrimination accuracy using the RF method combined with FD pretreatment to distinguish non-transgenic and transgenic rice seeds. This further indicated that FD-t-SNE-RF had a more substantial generalization and robustness for rice identification. Therefore, in this study, the best model for rice classification was FD-t-SNE-RF.

### 2.5. RF Classification Model Optimization

RF is different from the traditional decision tree algorithm, which has the characteristics of avoiding data overfitting without pruning, as well as a faster training speed and simple parameter adjustment, and has a better classification modeling effect under the default parameters [35]. In the RF algorithm, parameters such as the pruning threshold, the number of decision tree trees, and the number of categorized rice samples have a particular impact on the output of the RF classification model.

Regarding the hyperparameter optimization problem of machine learning classification models [36], the related literature primarily focuses on finding the optimal hyperparameters by using forward search methods or grid search methods [36,37], which are time-consuming or miss the optimal values due to the improper selection of the search step size [36]. This study used the intelligent optimization algorithm GWO [38] to optimize two hyperparameters (maximum tree growth depth and the number of hyperparameter subtrees) in the RF classification model. The dimension was set to 2 in the optimization process, and *p* and *m* had an optimization ranges of [1, 200] and [1, 30], respectively. These ranges impose constraints on the GWO algorithm during the optimization process. Subsequently, a cost function was developed to represent the objective function that needed to be minimized. Minimizing this cost function determines the optimal values of *p* and *m* that produce the maximum accuracy for rice classification. The final classification model accuracy using the GWO-optimized FD-TSNE-RF method is 95.3%. As it can be seen from Table 3, there is a 2% improvement in accuracy compared to the original unoptimized RF model. This indicates that the unoptimized RF classification model is potentially difficult to perform. The parameter optimization of GWO improves the model performance [39]; therefore, parameter optimization is necessary to build a high-accuracy rice variety classification model. This result is similar to that of Qiong Cao et al. [40], who achieved the best performance with the GWO-optimized SVM model for the classification of oolong tea varieties with multispectral information and the classification of the germination stage, with similar classification results. This also further indicates that the GWO-optimized RF rice classification and identification model can perform well.

**Figure 8:**
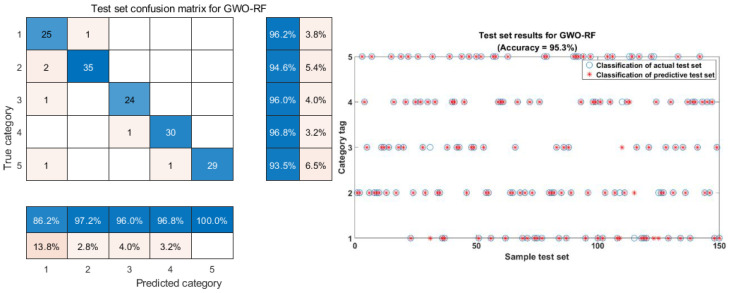
Classification results of the FD-TSNE-GWO-RF model test set.

Figure 8 shows the classification results of the FD-TSNE-GWO-RF model test set. From Figure 6, Figure 7 and Figure 8, it can be observed that the unoptimized RF classification model test set of 150 samples has 10 samples that are misclassified. In comparison, the GWO-optimized RF classification model has only seven samples that are misclassified, and the precisions of the optimized RF classification model for the five categories are all above 93.5%. This indicates that the established FD-TSNE-GWO-RF model has a more robust classification performance for rice identification. In summary, GWO makes the RF classification model advantageous for performance improvement. Therefore, this study finally determined the FD-TSNE-GWO-RF model as the best model for rice variety identification.

## 3. Materials and Methods

### 3.1. Sample

The rice varieties selected in this study were Thai jasmine rice, which is of good quality, aromatic, popular among consumers in different countries, and expensive in the market [41], and four types of comparatively cheaper Chinese domestic rice: Northeast Wuzhang rice, Northeast long-grain fragrant rice, Shaanxi Hanzhong rice, and Sichuan Meishan rice. These five types of rice were very similar in appearance and profile. The five types of rice selected for this experiment were all sourced from the same rice company. In order to ensure the representativeness of the experimental results, ten different production batches and dates were selected for each type of rice. Each sample was randomly sampled with 110 grains, totaling 5 × 110 single-grain rice samples. To ensure the accuracy of the samples, a rice expert from Ya’an was invited to authenticate the types of rice. The validated rice was sent to the laboratory for fluorescence hyperspectral data collection. JUN SUN et al. [42] used 60 samples from each of the four different regions of China purchased from the local Walmart supermarket in Zhenjiang, totaling 4 × 60 single-grain rice samples that were manually collected and scanned with a VIS-NIR hyperspectral imaging system, which ultimately yielded the highest identification accuracy (91.67%). This further demonstrates the scientific validity of the sample selection in this study.

### 3.2. Fluorescence Hyperspectral Image Acquisition

The fluorescence hyperspectral data of the rice samples were obtained using the GaiaFluo(/Pro)-VN-HR fluorescence hyperspectral test system produced by Sichuan Shuanglihe Spectrum Technology Co. (Chengdu, China) [20]. This fluorescence hyperspectral camera has the advantages of high sensitivity and strong signal [20]. The fluorescence hyperspectral resolution was 2.8 nm, and the pixel size was 2048 × 946 [43]. In this system, a xenon lamp is used as the excitation light source for the fluorescence imaging system, and the fluorescence hyperspectral range of the system can be detected from 250 nm to 1100 nm. The selection of filters is crucial during fluorescence hyperspectral image acquisition. By combining multiple excitation and fluorescence filters, it was found that, under the irradiation of four different excitation light source bands, the 390 nm excitation filter can better truncate the light input from other bands. Under the influence of the excitation light source, the fluorescence signal of the sample needs to be paid attention to. A 475 nm fluorescence filter can complete the separation of the fluorescence signal and parasitic light so that the final sample captured by the fluorescence hyperspectral camera produces the best fluorescence signal. Xiaohui Wang et al. [44] quantitatively predicted the non-destructive pH of kiwifruit by using the fluorescence hyperspectral imaging technique, and the prediction results were good. This is further evidence of the reliability of the choice of acquisition device in this study.

**Figure 9:**
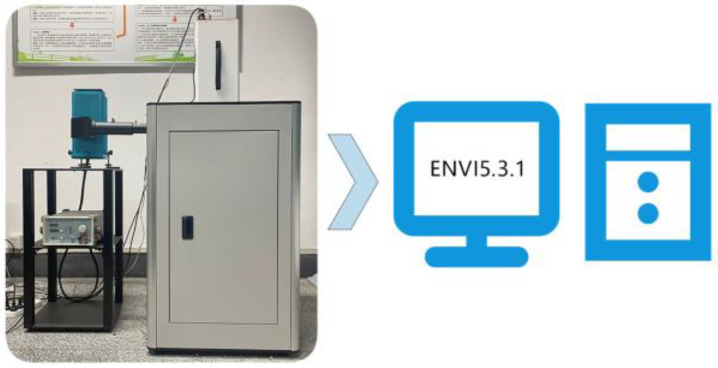
Fluorescence hyperspectral system.

The experiment was conducted at an ambient temperature of 26 °C and 50% ambient humidity. The RGB channels of the acquired fluorescence images were 341.2, 680.7, and 524.3, and the system movement speed was 0.26 mm/s with a camera exposure time of 800 ms. The fluorescence hyperspectral acquisition system is shown in Figure 9. Affected by the fluorescence filter, the final fluorescence hyperspectral range of the acquisition was from 376 nm to 1000 nm, with a total of 125 fluorescence hyperspectral channels. One hundred and ten rice samples from each variety were placed on a 10 × 11 counting plate, and then the plate was placed on a mobile carrier for the imaging test. A total of 5 × 110 single-grain rice samples were collected for the test.

### 3.3. Fluorescence Hyperspectral Data Extraction

**Figure 10:**
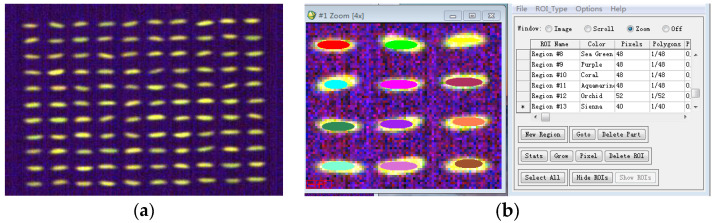
Fluorescence hyperspectral data extraction. (**a**) Fluorescence hyperspectral image of rice. (**b**) Data extraction of the fluorescence hyperspectral image of rice in the ENVI 5.3.1 software.

The ENVI5.3.1 software is a widely used remote sensing image processing tool [45]. After the acquisition and imaging with the fluorescence hyperspectral device, the image data are saved in the .raw and .hdr formats and must be imported into the ENVI5.3.1 software for subsequent processing [45]. The fluorescence hyperspectral image of the rice imported into the ENVI5.3.1 software is shown in Figure 10a. Since each pixel point in the image contains a fluorescence hyperspectral curve, the rice sample’s region of interest (ROI) [45] can be selected using the ENVI5.3.1 software. Region of interest extraction is to extract the target region in the fluorescence hyperspectral image of the rice sample. As shown in Figure 10b, in this study, the region of interest was extracted manually using ENVI5.3.1 in an elliptical manner according to the contour of the rice sample (the different colored parts in the figure are the manually selected ROIs) in the software, and the average fluorescence hyperspectral of all pixel points in the region of interest were taken as the fluorescence hyperspectral information of the rice samples. The extracted data represented the whole rice sample, reflecting the sample information. Deng Wei et al. [45] utilized hyperspectral imaging technology to select ROI-extracted data using the ENVI5.3.1 software and accurately identified cabbage seedlings and weeds using the SAM identification method. This further illustrates the rationality of this method.

### 3.4. Fluorescence Hyperspectral Data Preprocessing

Fluorescence hyperspectral data contain not only the chemical information of the sample to be measured but also random and systematic interference information (e.g., noise, stray light, light scattering, detector nonlinearity, and temperature variation) [46]. These interference signals complicate the fluorescence hyperspectral information and, in some cases, even mask the information of the components to be measured, thus most likely affecting the performance of the classification model. Therefore, the appropriate preprocessing of the fluorescence hyperspectral data before performing the analysis is significant for building models with a good predictive performance and high robustness [43].

In this study, mean centering (MC) [47,48], Savitzky–Golay convolutional smoothing (SG) [48], first-order derivative (FD), and standard normal variable (SNV) [47,48] were selected for preprocessing. The choice of preprocessing must be based on the specific distribution of the fluorescence hyperspectral data and the comprehensive judgment of the modeling performance after preprocessing.

MC can increase the difference between the fluorescence hyperspectral data of the samples and improve the robustness of the model [48]. The specific implementation of MC in this study was as follows.

(1) For the rice sample set’s spectral matrix (N×p), the average fluorescence hyperspectrum $\bar{x}$ of the rice sample was calculated.
  (1) $$\bar{x_{k}}=\frac{\sum_{i=1}^{N}x_{i,k}}{N}$$

N is the number of rice samples, k=1,2,…,p, and p is the number of wavelength points.

(2) We used spectrum x for the rice samples to obtain fluorescence hyperspectral data for the MC-treated rice.
  (2) $$x_{MC}=x-\bar{x}$$

SNV can effectively reduce the effects of baseline drift, tilt, and other noises [48]. The specific implementation of SNV in this study was as follows.

(3) $$x_{SNV}=\frac{x-\bar{x}}{\sqrt{\frac{\sum_{k=1}^{m}{\left(x_{k}-\bar{x}\right)}^{2}}{\left(m-1\right)}}}$$

x is the original fluorescence hyperspectrum of one of the rice samples, $\bar{x}$ is the average fluorescence hyperspectrum value of all rice samples, i=1,2,…,m, and m is the number of wavelength points.

The FD treatment can effectively process and reduce the effects of sample background and baseline drift and improve the resolution and sensitivity of the overlapping peaks.

FD was calculated in this study by the following formula.

(4) $$d_{FD}(i)=\frac{x(i+1)-x(i)}{\lambda (i+1)-\lambda (i)}$$

where x(i),(i=1,2,…,n) is the rice fluorescence hyperspectral sequence and λ(i),(i=1,2,…,n) is the fluorescence hyperspectral wavelength.

SG smoothing can improve the signal-to-noise ratio of the spectra and reduce the effects of random noise [48]. The formula followed for SG in this study was as follows.

(5) $$x_{i,k}(SG)=\frac{1}{H}\sum_{H}^{1}x_{i,k+j}h_{j},H=\sum_{j=\omega }^{+\omega }h_{j}$$

$x_{i,k}(SG)$ denotes the SG-smoothed value at the ith fluorescence hyperspectral wavelength, $x_{i,k+j}$ denotes the ith fluorescence hyperspectral value at the k+j wavelength, 2ω+1 denotes the width of the SG smoothing window, $h_{j}$ denotes the smoothing factor, and H denotes the normalization factor. The measurements are multiplied by the smoothing factor $h_{j}$ in order to minimize the effect of smoothing on the useful information.

### 3.5. Feature Downscaling and Feature Selection

The collected fluorescence hyperspectral data contain a large amount of redundancy, covariance, overlapping information, and a large amount of noise [49]. The original fluorescence hyperspectral data are selected to choose the wavelengths with the least covariance and redundancy and contain the primary valid information to reduce the interference of useless information [49]. These few or dozens of wavelengths are used to build the model instead of the original hundreds or even thousands of wavelengths to make the built model easier, more robust, and more accurate. In this paper, feature degradation and feature selection were performed using a principal component analysis (PCA), t-distributed stochastic domain embedding (t-SNE), linear discriminant analysis (LDA), and local linear embedding (LLE).

PCA [47] is a classical feature extraction algorithm. Currently, it is favored by a large number of scholars in spectral data processing [33]. PCA can solve the problem of data multicollinearity and extract data feature information to achieve data compression [33]. The principal component analysis transforms multiple variables to a new coordinate system by the null space of a linear transformation so that the first significant variance of any data projection is in the first coordinate (called the first principal component, PC1) [50]. The second significant variance is in the third coordinate (the second principal component, PC2), and so on, to obtain the same number of principal components as the number of variables [50]. These principal components are linear combinations of the original variables that are orthogonal to each other and contain no overlapping information, thus eliminating multicollinearity between the variables [33]. Theoretically, PCA obtains the same number of principal components with the exact dimensions as the original variable data, but since the contribution of the principal components is minimal and can be ignored except for the first few principal components that contribute the most to the variance, in practice, only a few principal components with the most significant contribution rate in the first part need to be retained in order to retain the essential information of the original data [33]. PCA can reduce the number of features, retain most of the efficient information, and be used in high-dimensional datasets [50]. PCA can be used for the exploration and visualization of high-dimensional datasets and can compress the existing features. Dimensionality reduction uses a data measure called sample variance or explainable variance [50]. The higher the variance, the more information the feature has [33]. The steps followed in this study to achieve rice fluorescence hyperspectral data compression and feature extraction were as follows.

(1) Normalize the rice fluorescence hyperspectral dataset.
(2) Calculate the covariance matrix of the standardized fluorescence hyperspectral data.
(3) Based on the covariance matrix, the principal component eigenvalues, principal component contribution rates, and cumulative contribution rates of the rice samples were calculated, and the principal component loadings were calculated.

Linear discriminant analysis (LDA) [51], which needs to consider the accurate labeling information of the training samples in the feature extraction process, is a classical supervised feature extraction method and is one of the most commonly used methods in spectral image feature reduction and extraction [51]. Compared with the PCA transform, the LDA transform seeks to find the optimal discriminant vector projection space for spectral data so that the interclass distance of the projected data is maximized and the intra-class distance is minimized [52], thus achieving the purpose of extracting class classification information and dimensionality reduction. The specific implementation process of LDA in this study was as follows. The detailed derivation and presentation of the LDA algorithm can be found in the paper [53].

(1) Input a fluorescence hyperspectral dataset $D=(x_{1},y_{1}),(x_{2},y_{2}),\ldots ,(x_{m},y_{m})$ of rice samples, where an arbitrary sample $x_{m}$ is an m-dimensional vector downscaled to dimension d.
(2) Calculate the intra-class scatter matrix of the dataset D.
(3) Compute the interclass scatter matrix for dataset D.
(4) Compute the new matrix: multiply the inverse of (2) by (3).
(5) Calculate the eigenvalues and eigenvectors of the matrix obtained from (4) and select the first d eigenvalues and the corresponding d eigenvectors in the order from smallest to largest to obtain the projection matrix W.
(6) New sample $z_{i}=W^{T}x_{m}\;\left(i=1,2,\ldots ,m\right)$.
(7) Output the fluorescence hyperspectral dataset of the rice samples $D^{\prime }=\left\{\left(z_{1},y_{1}),(z_{2},y_{2}),\ldots ,(z_{m},y_{m}\right)\right\}$.

Local linear embedding (LLE) is a popular learning method for classical nonlinear dimensionality reduction [54], which enables the reduced data to maintain its original topology with translational, rotational, and telescopic invariance and has an overall optimal solution without iteration, avoiding the problem of local extremes [54]. LLE considers each data point to be a linearly weighted combination of its neighboring points, and therefore, the basic steps of LLE in this study were as follows. For a detailed derivation and presentation of the LLE algorithm, please refer to reference [55].

(1) Assume first that the expression for the neighborhood linear relationship of the high-dimensional fluorescence hyperspectral data $x_{i}$ is expressed by.
  (6) $$x_{i}=w_{ih}x_{h}+w_{ik}x_{k}+w_{il}x_{l}$$
  where $w_{ih}$, $w_{ik}$, and $w_{il}$ are the weighting coefficients. Let the weighting coefficient be $w_{ij}$, which can be obtained by the following equation.
  (7) $$\left\{\begin{array}{c} \underset{w_{ij,j\in Q(i)}}{\min}\sum_{i=1}^{m}{\left.\left\|x_{i}-\sum_{j\in Q(i)}w_{ij}x_{j}\right.\right\|}_{2}^{\;2}\\ \sum_{j\in Q(i)}w_{ij}=1 \end{array}\right.$$
  where Q(i) denotes the high-dimensional fluorescence hyperspectral data set $x_{i}$ of the neighborhood data points and m denotes the number of rice fluorescence hyperspectral samples.
(2) Keeping Equation (7) unchanged, the low-dimensional space data point A can be obtained by Equation (8).
  (8) $$\underset{w_{ij,j\in Q(i)}}{\min}\sum_{i=1}^{m}{\left.\left\|y_{i}-\sum_{j\in Q(i)}w_{ij}y_{i}\right.\right\|}_{2}^{\;2}$$

T-SNE is a popular learning algorithm based on the stochastic nearest neighbor embedding (SNE) algorithm for visualizing high-dimensional datasets by representing them in a low-dimensional space of 2 or 3 dimensions [29]. t-SNE is a distribution of t-transformed values for individual samples, not for the overall samples, and it is an estimation of the distribution of the standard normal distribution of the values of the u-transformed variables [29]. The t-SNE dimensionality reduction algorithm in this study projects the rice samples in the high-dimensional space into the low-dimensional space while trying to preserve the local properties of the large sample set [56]. After t-SNE transforms the rice sample set, if it still has differentiability in the low-dimensional space, it indicates that the original rice sample set is differentiable; if it is presented as indivisible in the low-dimensional space, it may be because the rice sample set itself does not have differentiability, or the sample set is not suitable for projection to the low-dimensional space although it has the differentiability [56].The specific implementation process of t-SNE in this study was as follows. For a detailed derivation and presentation of the t-SNE algorithm, please refer to reference [57].

(1) Input the rice sample fluorescence hyperspectral dataset.
  (9) $$X=\left\{x_{1},x_{2},x_{3},\ldots ,x_{n}\right\},(n=1,2,3,\ldots ,i)$$
(2) The conditional probability distributions $x_{i}$ and $x_{j}$ between the two data points $p_{j|i}$ and $p_{i|j}$ are calculated using Equation (9), and the location information of the rice samples is represented by a Gaussian probability distribution.
  (10) $$p_{j|i}=\frac{\exp(-{\left\|x_{i}-x_{j}\right\|}^{2}/2\sigma_{i}^{2})}{\sum_{k\neq i}\exp(-{\left\|x_{i}-x_{k}\right\|}^{2}/2\sigma_{i}^{2})}$$
  (11) $$p_{i|j}=\frac{\exp({-\left\|x_{j}-x_{i}\right\|}^{2}/2\sigma_{j}^{2})}{\sum_{k\neq j}\exp(-{\left\|x_{j}-x_{k}\right\|}^{2}/2\sigma_{j}^{2})}$$
  In Equations (10) and (11), $\sigma_{i}$ is the variance of the Gaussian distribution corresponding to the rice sample data point $x_{i}$.
(3) Setting up the joint probability substep $p_{ij}$, obtain a low-dimensional sample random initial solution $y^{\left(0\right)}$.
  (12) $$p_{ij}=\frac{p_{j|i}+p_{i|j}}{2}$$
  (13) $$y^{\left(0\right)}=\left\{y_{1},y_{2},y_{3},\ldots ,y_{n}\right\}$$
(4) Calculate the joint distribution $q_{ij}$ of the low-dimensional sample space points in the t-distribution.
  (14) $$q_{ij}=\frac{{\left(1+{\left\|y_{i}-y_{j}\right\|}^{2}\right)}^{-1}}{\sum_{k\neq l}{\left(1+{\left\|y_{k}-y_{l}\right\|}^{2}\right)}^{-1}}$$
(5) Calculate the optimized gradient $\frac{\partial C}{\partial y_{i}}$.
  (15) $$\frac{\partial C}{\partial y_{i}}=4\sum_{j}(p_{ij}-q_{ij})(y_{i}-y_{j}){\left(1+{\left\|y_{i}-y_{j}\right\|}^{2}\right)}^{-1}$$
(6) Loop iterations of (14) and (15) until the fluorescence hyperspectral low-dimensional data of the rice samples are obtained.
  (16) $$y=\left\{y_{1},y_{2},y_{3},\ldots ,y_{n}\right\}$$

### 3.6. Machine Learning Classification Models

Three machine-learning classification algorithms were selected for this study. These methods have an excellent performance in classification models and high usage in other applications. The partial least squares discriminant analysis (PLS-DA) [58] method is a mathematical optimization technique in which the matrix refers to the category information of the samples in the form of a code during the construction of the model, and it has a very efficient discriminative ability by linear statistical modeling through the fluorescence hyperspectral information and the categories. The BP neural network [59] is based on the characteristics of the human brain’s neural network, which is mainly designed to simulate high-level brain functions through an artificial method. It helps to enhance the understanding of thinking and intelligence. Random forest (RF) consists of multiple individual decision trees combined with the bagging algorithm and randomization algorithm to construct a combination of decision makers, widely used in classification procedures [60]. Random forest (RF) is the most powerful machine learning algorithm.

#### 3.6.1. Partial Least Squares Discriminant Analysis (PLS-DA)

Partial least squares discriminant analysis (PLS-DA) [47] is a statistical method for classification and identification based on the PLS regression method [58]. In this study, the numerical variables were calibrated to different rice varieties, and a correction model was established to determine the different rice varieties by combining fluorescence hyperspectral features, which were calculated as follows [58].

(17) $$Y=X_{n\times p}B+E$$

*Y* is the response variable matrix; *n* is the number of rice samples; *p* is the number of fluorescence hyperspectral bands; *X* is the matrix of the fluorescence hyperspectral variable n×p; and *B* is the regression coefficients’ and residuals’ matrices of the fluorescence hyperspectral variables.

#### 3.6.2. BP Neural Network (BP)

The BP neural network algorithm (BP) is a multilayer forward neural network based on the error backpropagation algorithm [8], which is an artificial intelligence method different from the traditional methods, and its main idea is to divide the learning process into two stages: one is forward propagation and the other is error backpropagation [59]. The error after the output is used to estimate the error of the previous layer, and then this error is used to estimate the error of the previous layer, and so on; layer by layer, with backpropagation, the error estimates of all layers are obtained. This creates a process in which the error of the input layer is passed from level to level in the opposite direction [59]. Hence, the algorithm is also known as the error backpropagation algorithm, or BP for short. The main process of BP modeling in this paper was as follows.

(1) The five rice samples were divided into a training set and a test set according to the randomized division method; the training set was used for training the BP models, and the test set was used to test the trained BP models.
(2) The data in the training set were normalized so as to reduce the adverse effects caused by singular sample data in the original rice sample data.
(3) The purelin transfer function was selected, and the gradient descent method was used for training; the network parameters, such as hidden layers, number of training times, learning rate, learning accuracy, and weight threshold, were configured.
(4) The BP model was trained with the normalized training rice sample set until the BP model was output when the set parameters were met (the number of training times was reached or the learning accuracy was satisfied).
(5) The trained BP model was tested with the test set after the normalization process. If the predicted values of rice were within the allowed error range (0.001) from the ideal output, it meant that this rice classification BP model was valid; then, proceed to step (6), or otherwise return to step (3) for parameter modification and continue training.
(6) The test set was classified with the trained rice classification BP model and the results were output.

#### 3.6.3. Random Forest (RF)

Random forest (RF) is an integrated classification model formed by drawing different combinations of samples from the initial samples and constructing different decision trees for each set of samples through bagging (bootstrap aggregating) with the put-back sampling method [35]. Since there is a put-back sampling mechanism to perturb the data that produce the decision tree, the constructed decision trees are different and have a large variability, which improves the generalization of the overall model [35]. In classification tasks, voting decisions are usually made using the voting method to determine the final prediction results of multiple base models [60]. Among them, voting methods can be classified into three categories based on the voting method [60]:

(1) Absolute majority voting method that restricts the number of labeled votes to be greater than half of the votes.
(2) Relative majority voting method that decides the value simply based on the number of votes received.
(3) The weighted voting method is similar to the weighted average method. A suitable voting mechanism can lead to more accurate prediction results.

Random forests synthesize the prediction results of multiple decision trees with significant differences, which provides them the advantages of high prediction accuracy, fast convergence, and few regulation parameters [60]. After the research of Amaratunga et al. [61], the random forest method is one of the most excellent models among the current mainstream classifiers and integrators. The main steps of RF in this study were as follows. A detailed derivation and presentation of the RF algorithm can be found in reference [61].

(1) A subset of n–tree samples was extracted from the raw rice fluorescence hyperspectral data.
(2) A decision tree was generated using each sample subset, and at each node of the tree, variables were randomly selected to be split. The tree was grown continuously so that the number of nodes at each terminal node was not lower than the size of the node.
(3) A classification was developed using a voting mechanism to count the results of the n–tree decision tree.

### 3.7. Model Optimization Algorithm

The gray wolf optimization (GWO) algorithm is a bionic meta-heuristic fast search algorithm inspired by the prey-hunting behavior of gray wolves and proposed by Australian scholars Mirjalili et al. [39] for solving the practical engineering optimization problem of traditional non-convex surfaces [39]. The GWO algorithm simulates the hierarchical division of the gray wolf animals and the allocation of hunting tasks in the biosphere layer, which mainly includes the division of the leadership into a hierarchy and the allocation of the hunting process [38]. The algorithm divides gray wolves into α-wolves, β-wolves, δ-wolves, and w-wolves according to their leadership position in the hierarchy, arranged from top to bottom like a pyramid [38]. The predation task phase generally consists of three main hunting steps: dispersal to find prey, active encirclement of the prey, and active prey attack [39]. In this study, α-wolf, as the leader, must constantly communicate with the other three wolf species to find the minimum objective function of the RF model through information exchange and updates. In the GWO-optimized RF model in this paper, the main process was as follows. The detailed optimization principles of the GWO intelligent optimization algorithm can be found in reference [39].

(1) Input the rice sample data and normalize the data.
(2) Set the optimization range of rice classification RF model parameters and initialize the wolf pack and GWO parameters.
(3) Calculate the fitness value of the gray wolves and classify the wolves into four tiers, α, β, δ, and ω, by taking the root-mean-square error of the classification result as the fitness value.
(4) Update the positions of the wolves, recalculate the fitness values at the new positions, and re-elect the new α, β, and δ.
(5) When the number of iterations reaches the set maximum number of iterations, it indicates the end of training, and the optimal m (the maximum growth in depth of the tree) and p (the number of over-parameter subtrees) are output; otherwise, continue the parameter optimization.
(6) Use the optimal m and p to establish a rice classification model, test the test set, and output the results of the inverse normalization process.

### 3.8. Confusion Matrix

**Figure 11:**
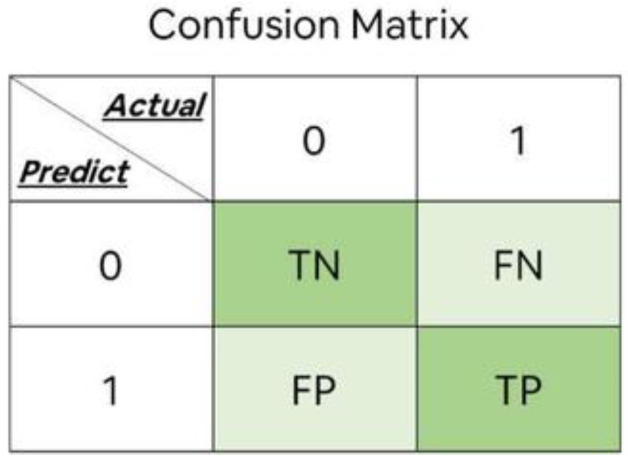
Schematic of the confusion matrix for the binary classification model.

Confusion matrix, or error matrix, measures how accurately a classifier classifies [34]. In calculating the multi-label confusion matrix, the multi-classification problem can be transformed into a binary classification, i.e., [62] a particular class has positive samples, and the rest are negative samples. From Figure 11 and Equations (18)–(20), it can be seen that Accuracy is the ratio of the number of correctly classified samples to the total number of samples, which measures the model’s ability to classify the samples, and the model accuracy is calculated from the confusion matrix. Precision is the ratio of the actual samples to the predicted positive samples. Recall is the ratio of true samples to the actual positive samples.

(18) $$Accuracy=\frac{TP+TN}{TP+FP+FN+TN}\times 100\%$$

(19) $$Precision=\frac{TP}{TP+FP}$$

(20) $$Recall=\frac{TP}{TP+FN}$$

In the formula, TP is the true case, that is, it is judged as a positive sample and is actually a positive sample; TN is the true counter-example, that is, it is judged as a negative sample and is actually a negative sample; FP is the false positive case, that is, it is judged as a positive sample but is actually a negative sample; and FN is the false counter-example, that is, it is judged as a negative sample but is actually a positive sample.

### 3.9. Evaluation Indicators

Accuracy is an essential metric in evaluating the performance of classification models in machine learning [63]. Equation (21) is the ratio of correct classifications to the total samples. The higher the model’s accuracy, the better the model’s classification. All machine learning algorithms in this paper were run on a CPU (Intel(R) Core (TM) i7-4510U CPU@2.60 GHz) and were implemented on MATLAB 2018b.

(21) $$Accuracy=\frac{“Number\;of\;correct\;classifications”}{“Total\;samples”}\times 100\%$$

## 4. Conclusions

The results of this study show that fluorescence hyperspectral techniques and machine learning can achieve the non-destructive detection and classification of rice varieties.

(1) FD preprocessing can effectively reduce the effects of sample background and baseline drift, improve the resolution and sensitivity of overlapping peaks, and improve the accuracy of the rice classification model.
(2) Feature dimensionality reduction and feature selection can reduce data redundancy and improve the prediction accuracy and robustness of the model. In comparing the four feature dimensionality reduction methods, selecting t-SNE had the best effect. t-SNE can retain most of the information in the rice spectral curve, effectively reduce the dimensionality of the spectral data of rice samples, and improve the robustness and generalization of the rice classification model.
(3) Among the three classification models, RF provided the best results. Combining RF with preprocessing and feature dimensionality reduction processing provided the best performance. Among them, the FD-t-SNE-RF method achieved 99.7% and 93.3% accuracy values for the training and test sets, respectively, and the classification model accuracy was higher than that of other spectroscopic and chemical methods. The RF classification model effectively identifies rice varieties accurately and has a superior reliability compared to other classification models.
(4) After the classification modeling of the data after preprocessing and feature dimensionality reduction, the parameters of the RF classification model were optimization-seeking after the introduction of the GWO optimization algorithm, and the optimal model for rice classification and identification was finally determined. Comparing the modeling results, it can be seen that the GWO optimization algorithm played a positive role in the classification of the RF model and improved the classification accuracy. The optimal classification model was FD-t-SNE-GWO-RF, and the training set accuracy and test set accuracy were 99.8% and 95.3%, respectively. This study demonstrates the superiority of the GWO optimization algorithm in rice classification model identification.
(5) The confusion matrix of the classification model shows that rice varieties with similar fluorescence hyperspectral information have specific errors in identification. Further machine-learning data processing is needed for rice variety identification analysis.

The experimental results show that fluorescence hyperspectral technology can achieve the identification of common rice varieties that are similar in appearance to Thai jasmine rice. As a new technology, fluorescence hyperspectral technology has the advantages of being non-destructive, efficient, and highly sensitive and providing real-time monitoring. Firstly, this study provided a simple and non-destructive detection method to identify whether Thai jasmine rice is adulterated in the market. It will help to improve the efficiency of rice detection in the Thai jasmine rice industry, enhance the Thai jasmine rice industry in areas where Thai jasmine rice is grown, and promote local economic development. Secondly, the model proposed in this study is of great significance for rapidly detecting rice adulteration in production lines, saving labor and testing costs, ensuring the authenticity of rice sales, standardizing the order of the rice market, and protecting the legitimate rights and interests of consumers. Finally, it provides a rapid and non-destructive detection method for other food safety identification fields.

## Figures and Tables

**Table 1:** Classification results of the various preprocessing methods for different models.

Methods	Models of Classification		
	BP	PLS-DA	RF
RAW	78%	79%	82%
MC	77%	78%	81%
SG	80%	79%	85%
SNV	82%	83%	88%
FD	84%	85%	91%

**Table 2:** Accuracy of the building classification models after using different preprocessing methods and feature dimensionality reduction processing methods.

Models	Methods	Training Accuracy	Test Accuracy
RF	FD-TSNE	99.7%	93.3%
	FD-PCA	96.2%	90.2%
	SNV-TSNE	90.7%	86.6%
	SNV-PCA	90.2%	88.5%
PLS-DA	FD-TSNE	91.2%	90.6%
	FD-PCA	90.5%	90.2%
	SNV-TSNE	90.2%	90.1%
	SNV-PCA	90.1%	90.0%
BP	FD-TSNE	95.5%	86.6%
	FD-PCA	90.2%	84.5%
	SNV-TSNE	90.1%	84.2%
	SNV-PCA	89.5%	82.2%

**Table 3:** Accuracy of classification prediction modeling using the FD-TSNE approach.

Methods	Training Accuracy	Test Accuracy
RF	99.7%	93.3%
GWO-RF	99.8%	95.3%

## Data Availability

The data presented in this study are available upon request from the authors.
